# Social media threats and health among adolescents: evidence from the health behaviour in school-aged children study

**DOI:** 10.1186/s13034-024-00754-8

**Published:** 2024-05-29

**Authors:** Henri Lahti, Marja Kokkonen, Lauri Hietajärvi, Nelli Lyyra, Leena Paakkari

**Affiliations:** 1 Faculty of Sport and Health Sciences, University of Jyväskylä, P.O. Box 35 (L), 40014 Jyväskylä, Finland; 2 Department of Education, University of Helsinki, Helsinki, Finland

**Keywords:** Social media threat, Adolescent, Health, Depressive feelings, Anxiety, Cyberbullying, Sexual harassment, Racism, Misinformation, Social media challenges

## Abstract

**Background:**

Social media are immensely popular among adolescents. Thus, concerns have been raised about the threats adolescents encounter on social media and the possible negative health consequences, such as depressive symptoms and anxiety. This study investigated the prevalence of nine social media threats: (1) cyberbullying, (2) sexual harassment, (3) racism, (4) unauthorized distribution of sensitive material, (5) phishing attempts, (6) misinformation, (7) the sale or distribution of drugs, (8) harmful or dangerous social media challenges, (9) content causing appearance pressures. The study also investigated how individual and social factors, problematic social media use (PSMU), and online communication with strangers are associated with social media threat exposure, as well as the association between social media threats and self-rated health, depressive feelings, and anxiety symptoms.

**Methods and findings:**

Nationally representative Health Behaviour in School-aged Children (HBSC) data from Finland were obtained from 2288 respondents aged 11, 13, and 15 years. Fixed effects regression models were applied. The most common threat, encountered daily and weekly, was misinformation. Regression models showed that individual and social factors, PSMU, and online communication with strangers explained adolescent exposure to social media threats in differing ways. Furthermore, certain factors (e.g., emotional intelligence, family support) were associated with encountering social media threats less frequently, whereas other factors (e.g., PSMU, online communication with strangers) were associated with more frequent encounters. Daily and weekly exposure to social media threats was systematically associated with poor self-rated health, frequent depressive feelings, and anxiety symptoms.

**Conclusions:**

Our study highlights the need for intervention and health promotion efforts to mitigate adolescent exposure to social media threats and ensuing negative health consequences.

## Background

During the past decade, the social media, including social networking sites and instant messengers, have gained immense popularity among adolescents [1]. The international study on Health Behaviour in School-aged Children [2] found that 41% of 15-year-olds used social media throughout the day, almost all the time. Moreover, research conducted on US adolescents aged 13–17 found that the number of young people reporting constant online presence almost doubled from 24% in 2015 to 45% in 2018 [3, 4]. Although social media may benefit adolescents by increasing social connectedness and fostering social self-identity [5], concerns have been raised about the threats encountered by young people on social media [6–8], and the ensuing unfavourable health consequences such as depressive symptoms and anxiety [9, 10]. Social media threats include a broad range of risky and harmful situations facilitated by the social media [11] such as cyberbullying [12], online discrimination (e.g., sexual harassment, racial discrimination) [9, 13], and misinformation [14].

There appear to be two notable reasons for the vulnerability of adolescents to social media threats. Firstly, adolescence represents a window of developmental sensitivity due to profound social, biological, and psychological development [15]. It is a widely held view that substantial changes in the adolescent social brain make adolescence a sensitive period for social interaction (involving, for example, more approaches to peers than among children aged < 10) [16], peer influence [17, 18], and self-perception [19]. Adolescence also represents a time of heightened susceptibility to risk-taking behaviour [20] and vulnerability [6]. In today’s world, the social media facilitate all these developmental processes [5]. Secondly, social media are particularly popular among young people. Adolescents report an increasing use of social media to socialize, share their lives, learn about their peers’ lives, explore their interests, search for information, and entertain themselves [21, 22]. This has led to many adolescent offline problems transferring to online contexts, and to the emergence of new threats. These are bound up with several social media features, notably the flow of rapidly spreading information [23] and the broad audience reach [14]. Ensuring adolescent safety on these platforms has therefore been incorporated as a key component of the European Strategy for a Better Internet for Kids [24] and the EU Strategy on the Rights of the Child [25]. Thus, exploring factors that could protect or place adolescents at risk of social media threats and how social media threats could harm adolescent health has become crucial for effective decision-making. The present study examined adolescent social media threats and their prevalence, together with the associated individual and social factors, and health outcomes.

### Social media threats among adolescents

The social media have brought about a new era of victimization, involving unique challenges and consequences. This is particularly evident in the phenomenon of cyberbullying, defined broadly as bullying via electronic means [26, 27]. Social media allow perpetrators to target their victims at any time and any place (either in front of large audiences or privately) via 24/7 accessibility [28]. Additionally, the possibility of remaining anonymous on these platforms may embolden perpetrators to continue bullying with no fear of repercussions [29]. The absence of face-to-face cues on social media hides the negative consequences of cyberbullying [30], and without this critical feedback, aggressive behaviour may be more likely to recur [12, 31]. In addition, continuous exposure to online aggression can lead individuals to view this behaviour as more acceptable through reinforcement and role modelling, especially if it is rewarded socially [12, 32]. According to a recent systematic review, the prevalence of cyberbullying victimization ranges between 14% and 58% among adolescents [33]. The authors note that one reason for the inconsistency in the prevalence rates is the differences in recall periods across studies (lifetime, last year, last month, etc.); hence, more nuanced research is needed to determine how often adolescents are exposed to cyberbullying [33].

Discrimination via social media, which includes online sexual harassment [9] and racial discrimination [13], presents another significant threat to adolescents. Previous studies have primarily defined online sexual harassment as unwanted sexual behaviour that occurs electronically, such as sending unsolicited sexual messages, images, or requests, or having sexual messages or images shared without permission [9, 34]. Online racial discrimination, on the other hand, refers to any unfair or prejudicial online act based on, for example, race, skin colour, or ethnicity [13]. Noting the easily accessible nature of social media (such that anyone can approach anyone), the rapid dissemination of information, and the broad audience reach, studies have suggested that social media have increased encounters with both forms of discrimination, beyond parental oversight [8]. Social media platforms may amplify discrimination by allowing perpetrators to target multiple victims simultaneously, while remaining anonymous and maintaining a physical distance [9, 35]. Like cyberbullying, the prevalence of online sexual harassment has been inconsistent across adolescent studies (1–59%), partly due to differences in recall periods [36]. The literature on racial discrimination, for its part, has mainly focused on vulnerable populations (e.g., adolescents of colour) [13] or adults [35]. Consequently, the prevalence of adolescents’ encounters with racial discrimination remains relatively unknown. One of the few studies on this issue found that out of adolescents aged 12–16, 17% reported at least monthly exposure to hate messages attacking certain groups or individuals [8].

One social media threat related to both cyberbullying and discrimination – as shown in previous studies [e.g., 37] – is unauthorized sharing of sensitive material (e.g., sexually explicit images). Such material can circulate through adolescent social networks and can be difficult to eradicate from the web [37]. Sensitive material may be shared willingly (e.g., in a relationship), and later disseminated nonconsensually as an act of revenge (e.g., during breakup) [34]. Furthermore, sensitive material can be acquired through phishing attempts by third parties [38]. In both instances, the sensitive material can be used to blackmail the victim [34, 39]. In 2020, 11% of adolescents aged 9–16 had experienced personal data misuse, such as somebody using personal information in a way the victim did not like, or somebody using a person’s password to access personal information over the past year [8]. Notably, 20% of adolescents did not know how to change their privacy settings [8], a factor that may place adolescents in a vulnerable situation in terms of privacy.

According to Southwell et al. [14], the current generation of adolescents faces a massive proliferation of rapidly spreading misinformation, i.e., inaccurate or misleading information running contrary to the best scientific evidence [14, 40]. The concern over misinformation was predominantly evident during the COVID-19 pandemic, when a wave of information on the COVID-19 virus spread, especially on social media [23]. Furthermore, a large-scale European study has indicated clear disparities in capabilities to access valid information, with 41% of adolescents reporting an inability to assess the validity of online information [8].

Concerns have also been raised about exposure to risky online content, such as the sale, distribution, and misuse of drugs [41–43]. With increased societal restrictions and parental surveillance over access to most substances, the social media may provide a novel environment for the display of adolescents’ risk-taking proclivities [44, 45]. For example, initial evidence points toward the increased use of social media to buy and sell drugs; in this case, the media facilitate easy access to groups where products are distributed and allow contactless deliveries to end-consumers [46]. Social media also foster participation in risky social media challenges [43]. According to previous theories and models, such as the ‘super peer’ theory [47], and the Facebook Influence Model [41], the social media context amplifies peer influence, due to the increased volume of content portraying risky behaviours, and the quantifiable reinforcement of such behaviour in the forms of ‘likes’ and comments. The social media context may also exacerbate sensation-seeking by framing risky challenges as exciting and pleasurable [42]. In Europe, on average, 8–17% of adolescents aged 12–16 report that they have faced harmful content online at least monthly. Approximately 10% of adolescents mentioned having viewed content on experiences of taking drugs, how to commit suicide, or how to physically harm oneself [8].

Popular adolescent social media platforms contain an abundance of appearance-focused content [48]. This content tends to promote muscular and athletic ideals for males, such as large biceps, a V-shaped torso, and visible abs. For females, it endorses thin and curvaceous ideals, such as a lean physique, low body fat, and a thin waist with a prominent bottom or bosom [49]. One important way in which the social media differ from traditional media is that the content is user-generated [48]. Adolescents report spending considerable time and effort on their images to represent the ‘best’ version of themselves, a process which is enhanced by in-built photo-editing tools [50]. Adolescents are thus exposed to idealized and possibly edited content of people, including peers and influencers, through their use of social media [48]. Furthermore, 12% of people aged 12–16 had come upon content on ‘ways to be very thin’ at least monthly [8].

### Factors related to social media threats

There is a growing consensus among researchers that adolescent exposure to social media threats is a complex phenomenon with several explanatory factors. Factors such as gender, age, and capabilities play a role in the threats to which adolescents are exposed. For example, girls are more likely to report online sexual harassment [10] and to encounter appearance-focused social media content [51, 52], whereas boys seem to be more likely to seek out violent material [8], and to have a lower perception of the risks related to publication of data and photographs [53]. Adolescent age has further been hypothesized as explaining social media threat exposure, on the grounds that periods of increased sensitivity to certain threats are likely to occur in relevant developmental windows [15, 54]. For example, Smahel et al. [8] found that adolescents aged 15–16 are more likely than those aged 9–11 to encounter threats such as cyberbullying and personal data misuse. In addition, disparities in capabilities to protect oneself from social media threats have been identified [8]. Emotional intelligence, defined as the ability to identify and comprehend one’s own emotions as well as the emotions of others, and to utilize this understanding to effectively regulate one’s own behaviour and relationships [55], has been suggested as a protective factor against social media threats [56, 57].

In addition, social factors such as family affluence, family support, and friend support have been linked to adolescent social media threat exposure. Studies on young people have found those with lower socioeconomic status to be more likely to report negative social media experiences, such as receiving hurtful messages, or having pictures/videos shared without their consent [58, 59]. Conversely, higher family support has been found to significantly reduce adolescents’ risk of encountering social media threats. For example, a review study by Elsaesser et al. [60] found that higher family support was consistently associated with lower cyberbullying victimization and perpetration. The relationship between friend support and social media threats, however, is expected to be double-edged. On the one hand, a higher level of friend support has been shown to protect young people from social media threats such as cyberbullying [61]. On the other hand, friend support can enhance the likelihood of encountering and participating in risky behaviours (e.g., dangerous social media challenges) [43].

Research also suggests that social media–related disorders contribute to exposure to social media threats. For example, *problematic social media use* (PSMU) – as indicated by addiction-like symptoms (i.e., withdrawal, conflict, preoccupation) – has been associated with cyberbullying [12], and exposure to misinformation/misconceptions on COVID-19 during the outbreak [62]. The idea of PSMU being associated with social media threats is supported by social theories such as the Problem Behaviour Theory, which suggests that certain risk behaviours are interconnected and contribute to vulnerability [63, 64]. Concerns have also been raised about adolescents who use social media to intensively communicate with strangers. Although uncommon, strangers may pursue ill intentions such as solicitation and harassment [65]. Adolescents may be unable to identify malicious intents due to their sensitivity to acceptance, feelings of rejection, and a lack of self-awareness [5].

### Social media threats and health

Adolescents have experienced a significant increase in depression and anxiety over the last decade, and the social media have been suspected as a primary cause [66, 67]. However, the evidence is conflicting. On the one hand, numerous reviews have established a connection between social media use and negative health outcomes among young people [e.g.,^68^, ^69^]. On the other hand, a recent umbrella review concluded that the association between social media use and poor adolescent wellbeing was ‘weak’ and ‘inconsistent’ [70]. There have been calls for research to shed light on these inconsistent findings, focusing on the mechanisms that could make social media harmful to adolescents’ health [67, 70–73]. Encounters with social media threats have been proposed as one such mechanism [74]. Thus far, studies among young people have found cyberbullying, sexual harassment victimization, and racial discrimination to be associated with negative health outcomes such as depressive symptoms and anxiety [9, 10, 75, 76]. Misinformation can negatively influence adolescents’ health and health behaviour by eroding their judgement, and by shaping the precursors of their intentions [14]. These can include their attitudes toward behaviour, for example, in terms of approving or disapproving [14]. It may further disrupt their feelings of security, as has happened, for instance, via content related to COVID-19 [77]. Hence, misinformation has been associated with negative moods, anxiety, and distress [77, 78]. Furthermore, threats such as alcohol-related content, and harmful social media challenges have been related to harmful behavioural choices [14, 41, 42]. Idealized appearance-focused content on the social media, for its part, provides adolescents with opportunities to internalize prescriptive ideals, self-objectify, and engage in negative upward appearance comparisons, which could trigger body dissatisfaction [48, 79]. All this would suggest that encounters with social media threats would be a stronger determinant of negative health among adolescents than social media use alone [73]. 

### The current study

The current state of research leaves gaps in our understanding. We lack a comprehensive understanding of how frequently adolescents are exposed to various social media threats in Finland, or within the broader empirical context. There has been relatively little research on the prevalence of certain threats (e.g., dangerous social media challenges, or the sale or distribution of drugs), and the prevalence rates for certain threats (e.g., cyberbullying and sexual harassment) have been inconsistent across studies due to varying reporting frequencies [33, 36]. Some studies have explored the association between individual and social factors and social media threats under specific conditions; however, there has so far been no comprehensive examination of a broad set of individual and social factors in relation to various social media threats [6–8]. Furthermore, despite a recent surge in studies on social media use and health, our understanding of the mechanisms through which social media use might harm adolescent mental health and wellbeing remains limited [67, 70–73]. This emphasizes the need to determine how various social media threats are associated with health outcomes in adolescence. To address these research gaps, the present study aimed to evaluate adolescents’ encounters with nine social media threats at distinct intervals: ‘never’, ‘monthly’, ‘weekly’, and ‘daily’, and their association with individual and social factors, PSMU, online communication with strangers, and health outcomes. Thus, by utilizing a nationally representative sample of Finnish adolescents, the following research questions were addressed:

(RQ1) How prevalent are social media threats (cyberbullying, sexual harassment, racism, unauthorized distribution of sensitive material, phishing attempts, misinformation, sale or distribution of drugs, harmful or dangerous social media challenges, content causing appearance pressures)?

(RQ2) What are the associations between exposure to social media threats and (i) individual factors (gender, age, emotional intelligence), (ii) social factors (family affluence, family support, friend support), (iii) PSMU, (iv) online communication with strangers?

(RQ3) How are social media threats associated with health (self-rated health, depressive feelings, anxiety)?

Based on previous research, the following hypotheses were formed:

#### H1

 We expected the prevalence of social media threats among adolescents to vary depending on the threat type and the reporting frequency (i.e., never, monthly, weekly, daily). Misinformation was expected to be the most prevalent social media threat on a daily and weekly level, followed by content causing appearance pressures and harmful social media challenges.

#### H2

 Individual and social factors, PSMU, and online communication with strangers were expected to differently explain exposure to social media threats (H2.1). Emotional intelligence and family support were expected to protect adolescents from encountering social media threats, whereas (H2.2) PSMU and online communication with strangers were expected to increase vulnerability to social media threats.

#### H3

 Social media threats were expected to be associated with negative health outcomes, with the associations varying between different social media threats. The association between exposure to a social media threat and negative health outcomes was expected to increase as the prevalence of the exposure increased (i.e., never, monthly, weekly, daily).

## Methods

### Sample and procedure

Nationally representative data were collected from Finnish adolescents in 2022 as part of the international Health Behaviour in School-aged Children Study (HBSC). The data were collected through anonymous voluntary standardized questionnaires administered to young people aged 11, 13, and 15 via school-based surveys. A stratified random cluster sampling design was used, and the data collection followed guidelines prescribed by the HBSC research protocol [80]. Ethical approval for the study procedures was obtained from the institutional ethics committee of the University of Jyväskylä.

In total, the sample comprised of 2288 Finnish boys (*n =* 1117; 48.8%) and girls (*n =* 1171; 51.2%) between the ages of 11 (*n =* 904; 39.5%), 13 (*n =* 764; 33.4%), and 15 (*n =* 620; 27.1%).

### Measures and variables

#### Social media threats

*Social media threats* were measured via options covering nine social media threats. Respondents were asked to indicate how often they had encountered cyberbullying, sexual harassment, racism, unauthorized distribution of sensitive material, phishing attempts, misinformation, the sale or distribution of drugs, harmful or dangerous social media challenges, and content that causes appearance pressures. The response options ranged from 1 (daily) to 5 (never). The response options 2 (more than once a week), and 3 (at least once a week) were combined to represent weekly exposure. The items were then reverse scored: 1 = never, 2 = weekly, 3 = monthly, 4 = daily exposure. The social media threats were based on a Delphi study by Lahti et al. [74].

#### Individual factors

*Gender* (boy, girl) and *age* (11, 13, 15) were studied by asking respondents to choose the correct alternative [2, 80].

*Emotional intelligence* was measured using the 10-item Brief Emotional Intelligence Scale [81]. Respondents were asked to indicate if they knew why their emotions changed, if they could easily recognize their emotions as they experienced them, if they could tell how people were feeling by listening to their tone of voice, or by looking at their facial expressions, if they recognized the emotions people were experiencing, if they sought out activities that made them happy, if they had control over their emotions, if they arranged events that others enjoyed, if they helped other people to feel better when they were in low spirits, if they were able to come up with new ideas when in a positive mood, and if they used good moods to make themselves keep trying in the face of obstacles. The response scale ranged from 1) ‘describes me very poorly’ to 5) ‘describes me very well’. A mean score (range 0–5) was calculated from the items to indicate adolescent emotional intelligence. The scale has been validated and found reliable [81]. The Cronbach alpha of the composite score was 0.89, exceeding the Cronbach alpha coefficient found by Aronen et al. [82] using a small sample of 51 Finnish adults.

#### Social factors

*The Family Affluence Scale III* (FAS) [83] was used to measure the family’s socioeconomic position. The respondents were asked about the family’s ownership of a car, the family’s ownership of a dishwasher, having one’s own bedroom, number of family computers, number of family bathrooms, and number of family vacations during the past 12 months. A sum score was calculated from the items to indicate family affluence, in line with the suggestions of Elgar et al. [84]. The FAS III has been validated and shown to be appropriate in adolescent studies [83].

*Family support* was measured via Zimet et al.’s [85] Multidimensional Scale of Perceived Social Support. Respondents were asked to indicate whether ‘my family really tries to help me’, ‘I get the help and emotional support I need from my family’, ‘I can talk about my problems with my family’, and ‘my family is willing to help me in decision-making’. The response options ranged from 1 (very strongly disagree) to 7 (very strongly agree). A mean score (range 0–7) was calculated and used to indicate family support. The scale has been validated [86, 87], and has shown good reliability (Cronbach’s alpha 0.96).

*Friend support* was measured via Zimet et al.’s [85] Multidimensional Scale of Perceived Social Support. Respondents were asked to indicate whether ‘my friends really try to help me’, ‘I can count on my friends when something goes wrong’, ‘I have friends with whom I can share my joys and sorrows’, ‘I can talk about my problems with my friends’. The response options ranged from 1 (very strongly disagree) to 7 (very strongly agree). A mean score (range 0–7) was calculated and used to indicate friend support. The scale has been validated [86, 87], and has shown good reliability (Cronbach’s alpha 0.96).

#### PSMU and online communication with strangers

*Problematic social media use* was measured via nine items of the Social Media Disorder Scale [88, 89]. Respondents were asked whether they, in the past year, regularly could not stop thinking about social media (preoccupation), felt dissatisfied because they wanted to devote more time to social media (tolerance), often felt bad when they were unable to use social media (withdrawal), failed in efforts to reduce time spent on social media (persistence), regularly neglected doing other things because of social media (displacement), regularly had arguments with others because of their use of social media (problem), regularly lied to parents or friends about how much time they spent on social media (deception), often used social media to escape from negative feelings (escape), and had severe conflicts with parents or siblings because of their use of social media (conflict). The response options were 1 ‘yes’ and 0 ‘no’. Respondents who answered positively to 6–9 items were classified as 2 = problematic user, while the rest were classified as 1 = non-problematic user [88–90]. The scale has been found to be valid and reliable [88]. The internal consistency of the scale was adequate (Cronbach’s alpha 0.82).

*Online communication with strangers* was assessed using an item adapted from the EU Kids Online Survey [91]. Respondents were asked how often they had online contact through social media with unknown people. The responses ranged from 1 (never/almost never) to 5 (almost all the time throughout the day), with also a ‘do not know/does not apply’ option. Respondents answering with option 5 were categorized as 2 = having intensive online communication with strangers, whereas the respondents answering with options 1–4 were categorized as 1 = not having intensive communication with strangers. The categorization was based on previous studies utilizing the same item with different demographics such as close friends [e.g., 2, 92–94].

#### Health

*Self-rated health* (SRH) was measured via a single question on the individual’s evaluation of their health [95]. The response options were *poor, fair, good*, and *excellent*. Respondents who answered good and excellent were classified as having 1 = good SRH, whereas those answering fair and poor were classified as having 2 = poor SRH [see e.g., 96]. SRH has been shown to be a robust item [97], and valid in adolescent samples [98].

*Depressive feelings* were measured as part of the HBSC symptoms checklist [99]. The respondents were asked how often they had experienced depressive feelings over the last six months. The response options ranged from 1 (rarely or never) to 5 (about every day). Those having depressive feelings rarely or never or monthly were classified as 1 = not having depressive feelings frequently. Those having depressive feelings about every week, more than once a week, and about every day were combined and classified as 2 = having depressive feelings frequently. The item has been validated in an adolescent sample and has been found to have adequate reliability [100].

*Anxiety* was measured as part of the HBSC symptoms checklist [99]. The respondents were asked how often they had experienced anxiety over the last six months. The response options ranged from 1 (rarely or never) to 5 (about every day). Those having anxiety symptoms rarely, never, or monthly were classified as 1 = not having anxiety symptoms frequently. Those having anxiety symptoms about every week, more than once a week, and about every day were classified as 2 = having anxiety symptoms frequently.

### Statistical analyses

Missing data ranged between 1.4% (gender) and 15.6% (problematic social media use). To overcome the potential bias associated with listwise deletion, we utilized multiple imputation by chained equations. Multiple imputation reduces the potential bias related to missing data even when the percentage of missing data is high [101]. The missing data were imputed on the basis of available data on other included study variables. Five imputations were conducted, in line with the suggestions of Madley-Dowd et al. [101]; thus, all 2288 respondents were retained for the analyses.

The associations between *individual and social factors*, *PSMU, online communication with strangers*, and *social media threats* were tested using fixed effects multinomial logistic regression analyses, and reported as odds ratios (ORs). For the social media threats, ‘Never’ was used as the reference category. A separate analysis of 15-year-olds was performed for *emotional intelligence*, as the variable was only measured in this age group. Variables were added to the models hierarchically, and adjusted effects were reported.

Fixed effects binary logistic regression analyses were conducted to study the association between social media threats and health outcomes. The regression models were performed on each health outcome separately, and the analyses were adjusted for gender, age, and family affluence. All fixed effects logistic regression models were tested for the clustering effect of schools in the data. The analyses were conducted with IBM SPSS Statistics 28.0 [102].

## Results

### The prevalence of social media threats

As shown in Table 1, the two most prevalent social media threats encountered by adolescents daily were *misinformation* (12.9%) and *content causing appearance pressures* (9.1%). On a weekly basis, the most prevalent social media threats were *misinformation* (44.2%) and *harmful or dangerous social media challenges* (22.3%). In terms of monthly exposure, the most prevalent social media threats were *unauthorized distribution of sensitive material* (27.7%) and *harmful or dangerous social media challenges* (26.8%). The least prevalent social media threats (in terms of the ‘never encountered’ option) were *cyberbullying* (79.5%) and *sexual harassment* (77.7%).

**Table 1:** Prevalence of social media threats

	Daily	Weekly	Monthly	Never	Total	Significance	
	%	%	%	%	(n)	χ^2^	*p* value
Cyberbullying	2.8	6.8	10.9	79.5	2288		
Gender, girl	1.5	3.8	12.1	82.6	1171	50.79	< 0.001
Boy	4.1	9.9	9.7	76.3	1117		
Sexual harassment	3.0	7.6	11.7	77.7	2288		
Gender, girl	2.0	7.1	16.5	74.4	1171	59.90	< 0.001
Boy	4.1	8.2	6.6	81.1	1117		
Racism	6.3	18.4	19.1	56.2	2288		
Gender, girl	3.8	20.3	23.7	52.2	1171	62.34	< 0.001
Boy	9.0	16.4	14.3	60.3	1117		
Unauthorized distribution of sensitive material	5.6	22.2	27.7	44.5	2288		
Gender, girl	3.3	22.1	30.2	44.4	1171	27.59	< 0.001
Boy	7.9	22.4	25.0	44.7	1117		
Phishing attempts	4.3	12.5	20.7	62.5	2288		
Gender, girl	1.7	8.8	23.7	65.8	1171	76.77	< 0.001
Boy	6.9	16.3	17.6	59.2	1117		
Misinformation	12.9	44.2	25.6	17.3	2288		
Gender, girl	8.4	46.4	28.4	16.8	1171	49.65	< 0.001
Boy	17.8	41.9	22.6	17.7	1117		
Sale or distribution of drugs	8.5	18.2	13.4	59.9	2288		
Gender, girl	8.9	19.5	14.7	56.9	1171	8.96	0.054
Boy	8.2	17.0	12.0	62.8	1117		
Harmful or dangerous social media challenges	5.6	22.3	26.8	45.3	2288		
Gender, girl	3.3	22.1	32.9	41.7	1171	62.55	< 0.001
Boy	8.0	22.4	20.5	49.1	1117		
Content that causes appearance pressures	9.1	18.9	15.3	56.7	2288		
Gender, girl	13.3	25.9	19.6	41.2	1171	237.21	< 0.001
Boy	4.7	11.7	10.9	72.8	1117		

### The associations of individual factors with social media threats

As indicated by Table 2, significant associations were identified between social media threats and individual factors. In terms of *encountering social media threats daily*, seven out of the nine threats were more likely to be reported by boys, including cyberbullying (OR = 0.28, CI 95% = 0.15–0.53), sexual harassment (OR = 0.51, CI 95% = 0.27–0.96), racism (OR = 0.47, CI 95% = 0.31–0.70), unauthorized distribution of sensitive material (OR = 0.37, CI 95% = 0.24–0.57), phishing attempts (OR = 0.18, CI 95% = 0.11–0.32), misinformation (OR = 0.47, CI 95% = 0.34–0.66), and harmful or dangerous social media challenges (OR = 0.42, CI 95% = 0.27–0.65). By contrast, content causing appearance pressures (OR = 6.71, CI 95% = 4.51–9.98) was the only threat more likely to be reported by girls.

In terms of *weekly exposure*, cyberbullying (OR = 0.32, CI 95% = 0.21–0.48) and phishing attempts (OR = 0.43, CI 95% = 0.32–0.57) were more likely to be reported by boys, whereas exposure to racism (OR = 1.40, CI 95% = 1.10–1.79) and content causing appearance pressures (OR = 4.79, CI 95% = 3.65–6.29) were more likely to be reported by girls. Girls were also more likely to report *monthly* exposure to sexual harassment (OR = 2.53, CI 95% = 1.75–3.64), racism (OR = 1.89, CI 95% = 1.49–2.41), misinformation (OR = 1.34, CI 95% = 1.02–1.75), harmful and dangerous challenges (OR = 1.89, CI 95% = 1.53–2.35), and content causing appearance pressures (OR = 3.71, CI 95% = 2.84–4.84).

Adolescents aged 15 self-reported *daily* (ORs 2.82–20.89) and *weekly* (ORs 2.05–11.11) exposure to every social media threat more than did those aged 11 (Table 2). Similarly, compared to 11-year-olds, 13-year-olds were more likely to encounter six out of the nine social media threats *daily* (ORs 2.00–7.71), every social media threat *weekly* (ORs 2.20–5.21), and eight out of the nine threats *monthly* (ORs 1.42–3.12). Adolescents with higher emotional intelligence were less likely to report *daily* exposure to cyberbullying (OR = 0.40, CI 95% = 0.23–0.72), sexual harassment (OR = 0.34, CI 95% = 0.17–0.70), racism (OR = 0.57, CI 95% = 0.36–0.89), unauthorized distribution of sensitive material (OR = 0.57, CI 95% = 0.35–0.94), and phishing attempts (OR = 0.49, CI 95% = 029–0.84).

**Table 2:** The association of individual factors with social media threats

Variable	Gender (ref. boy)		Age (ref. 11y)				Emotional intelligence (continuous)	
			13y		15y			
	OR (CI 95%)	*p* value	OR (CI 95%)	*p* value	OR (CI 95%)	*p* value	OR (CI 95%)	*p* value
Cyberbullying (ref. never)								
Daily	**0.28 (0.15–0.53)**	**< 0.001**	1.62 (0.78–3.35)	0.198	**2.82 (1.38–5.76)**	**0.005**	**0.40 (0.23–0.72)**	**0.003**
Weekly	**0.32 (0.21–0.48)**	**< 0.001**	**2.24 (1.40–3.59)**	**< 0.001**	**2.05 (1.26–3.31)**	**0.004**	0.71 (0.44–1.15)	0.162
Monthly	1.10 (0.82–1.48)	0.517	1.02 (0.72–1.45)	0.892	1.12 (0.79–1.60)	0.519	0.89 (0.57–1.39)	0.605
Sexual harassment (ref. never)								
Daily	**0.51 (0.27–0.96)**	**0.038**	1.82 (0.83–4.01)	0.135	**3.72 (1.89–7.31)**	**< 0.001**	**0.34 (0.17–0.70)**	**0.005**
Weekly	0.91 (0.63–1.30)	0.601	**3.31 (1.98–5.53)**	**< 0.001**	**4.43 (2.66–7.36)**	**< 0.001**	0.73 (0.47–1.14)	0.169
Monthly	**2.53 (1.75–3.64)**	**< 0.001**	**2.03 (1.36–3.01)**	**< 0.001**	**4.24 (2.95–6.07)**	**< 0.001**	0.94 (0.61–1.43)	0.753
Racism (ref. never)								
Daily	**0.47 (0.31–0.70)**	**< 0.001**	**5.36 (3.06–9.38)**	**< 0.001**	**7.19 (4.15–12.45)**	**< 0.001**	**0.57 (0.36–0.89)**	**0.015**
Weekly	**1.40 (1.10–1.79)**	**0.006**	**3.04 (2.23–4.14)**	**< 0.001**	**3.52 (2.54–4.87)**	**< 0.001**	0.80 (0.55–1.15)	0.221
Monthly	**1.89 (1.49–2.41)**	**< 0.001**	**2.61 (1.98–3.45)**	**< 0.001**	**2.92 (2.19–3.90)**	**< 0.001**	1.21 (0.82–1.78)	0.336
Unauthorized distribution of sensitive material (ref. never)								
Daily	**0.37 (0.24–0.57)**	**< 0.001**	**3.15 (1.81–5.49)**	**< 0.001**	**5.86 (3.40–10.10)**	**< 0.001**	**0.57 (0.35–0.94)**	**0.026**
Weekly	0.91 (0.71–1.16)	0.436	**2.87 (2.13–3.88)**	**< 0.001**	**4.61 (3.41–6.23)**	**< 0.001**	0.92 (0.63–1.33)	0.640
Monthly	1.15 (0.92–1.44)	0.215	**2.05 (1.60–2.63)**	**< 0.001**	**3.04 (2.31–4.01)**	**< 0.001**	0.96 (0.65–1.41)	0.824
Phishing attempts (ref. never)								
Daily	**0.18 (0.11–0.32)**	**< 0.001**	2.07 (0.98–4.39)	0.057	**4.78 (2.44–9.38)**	**< 0.001**	**0.49 (0.29–0.84)**	**0.010**
Weekly	**0.43 (0.32–0.57)**	**< 0.001**	**2.20 (1.56–3.11)**	**< 0.001**	**2.79 (1.96–3.96)**	**< 0.001**	0.76 (0.49–1.17)	0.203
Monthly	1.16 (0.93–1.45)	0.194	**1.99 (1.49–2.68)**	**< 0.001**	**3.39 (2.59–4.46)**	**< 0.001**	1.09 (0.79–1.52)	0.601
Misinformation (ref. never)								
Daily	**0.47 (0.34–0.66)**	**< 0.001**	**3.29 (2.18–4.97)**	**< 0.001**	**5.62 (3.69–8.57)**	**< 0.001**	1.03 (0.64–1.65)	0.911
Weekly	1.12 (0.87–1.45)	0.378	**2.77 (2.07–3.70)**	**< 0.001**	**3.73 (2.62–5.31)**	**< 0.001**	1.22 (0.80–1.84)	0.356
Monthly	**1.34 (1.02–1.75)**	**0.033**	**1.42 (1.04–1.94)**	**0.028**	**1.53 (1.06–2.19)**	**0.022**	1.21 (0.73–2.01)	0.463
Sale or distribution of drugs (ref. never)								
Daily	1.08 (0.75–1.54)	0.691	**7.71 (4.27–13.94)**	**< 0.001**	**20.89 (11.86–36.80)**	**< 0.001**	0.98 (0.64–1.51)	0.928
Weekly	1.19 (0.90–1.56)	0.215	**5.21 (3.72–7.28)**	**< 0.001**	**11.11 (7.90–15.61)**	**< 0.001**	0.92 (0.65–1.31)	0.651
Monthly	1.25 (0.93–1.68)	0.133	**3.12 (2.24–4.33)**	**< 0.001**	**4.95 (3.51–6.98)**	**< 0.001**	0.82 (0.56–1.20)	0.303
Harmful social media challenges (ref. never)								
Daily	**0.42 (0.27–0.65)**	**< 0.001**	**2.00 (1.19–3.38)**	**0.009**	**4.24 (2.58–6.96)**	**< 0.001**	0.70 (0.45–1.10)	0.123
Weekly	1.13 (0.89–1.42)	0.319	**2.55 (1.90–3.41)**	**< 0.001**	**3.43 (2.53–4.64)**	**< 0.001**	0.85 (0.57–1.27)	0.421
Monthly	**1.89 (1.53–2.35)**	**< 0.001**	**1.92 (1.48–2.51)**	**< 0.001**	**2.58 (1.96–3.39)**	**< 0.001**	0.87 (0.59–1.28)	0.473
Content that causes appearance pressures (ref. never)								
Daily	**6.71 (4.51–9.98)**	**< 0.001**	**4.27 (2.70–6.78)**	**< 0.001**	**5.85 (3.64–9.42)**	**< 0.001**	0.62 (0.35–1.08)	0.088
Weekly	**4.79 (3.65–6.29)**	**< 0.001**	**3.55 (2.53–4.96)**	**< 0.001**	**5.68 (4.12–7.85)**	**< 0.001**	0.82 (0.57–1.17)	0.276
Monthly	**3.71 (2.84–4.84)**	**< 0.001**	**2.93 (2.14–4.01)**	**< 0.001**	**2.75 (1.97–3.83)**	**< 0.001**	0.87 (0.56–1.35)	0.522

Fixed-effect multinomial logistic regression models: odds ratios (OR); 95% confidence intervals (CI); *ref.* reference category.

The significance level was set at *p* < 0.05 . The significant associations have been **bolded**

Regression models for each social media threat were run separately. The models were adjusted for gender, age, emotional intelligence, FAS, family support, friend support, PSMU, online communication with strangers. Social media threats were treated as outcome variables in the models

Emotional intelligence was only included for 15-year-olds

### The association of social factors with social media threats

Adolescents with higher family affluence were more likely to report *daily* encounters with misinformation (OR 2.24, CI 95% = 1.25–4.03), sale or distribution of drugs (OR = 1.85, CI 95% = 1.03–3.35), and content causing appearance pressures (OR = 1.81, CI 95% = 1.00–3.29), *weekly* exposure to content causing appearance pressures (OR = 1.67, CI 95% = 1.05–2.65), *monthly* encounters with harmful social media challenges (OR = 1.76, CI 95% = 1.21–2.57), or content causing appearance pressures (OR = 2.63, CI 95% = 1.66–4.18; Table 3).

Adolescents with higher family support were less likely to report *daily* (ORs 0.60–0.78) and *weekly* (ORs 0.72–0.87) exposure to eight out of the nine social media threats, and *monthly* (ORs 0.78–0.86) exposure to three social media threats. In terms of social support from friends, adolescents with higher support were more likely to report *daily* encounters with the sale or distribution of drugs (OR = 1.19, CI 95% = 1.05–1.36), but less likely to report *daily* (OR = 0.78, CI 95% = 0.64–0.94) and *weekly* (OR = 0.75, CI 95% = 0.65–0.85) encounters with cyberbullying.

**Table 3:** The association of social factors with social media threats

Variable	Family affluence (continuous)		Family support (continuous)		Friend support (continuous)	
	OR (CI 95%)	*p* value	OR (CI 95%)	*p* value	OR (CI 95%)	*p* value
Cyberbullying (ref. never)						
Daily	1.32 (0.45–3.88)	0.605	**0.70 (0.57–0.86)**	**< 0.001**	**0.78 (0.64–0.94)**	**0.010**
Weekly	0.76 (0.38–1.53)	0.440	**0.82 (0.72–0.93)**	**0.003**	**0.75 (0.65–0.85)**	**< 0.001**
Monthly	1.08 (0.64–1.82)	0.770	0.92 (0.82–1.03)	0.131	0.91 (0.81–1.02)	0.088
Sexual harassment (ref. never)						
Daily	0.60 (0.22–1.61	0.304	**0.60 (0.49–0.72)**	**< 0.001**	0.94 (0.78–1.13)	0.507
Weekly	1.22 (0.54–2.79)	0.619	**0.72 (0.62–0.83)**	**< 0.001**	0.95 (0.83–1.09)	0.474
Monthly	1.00 (0.61–1.64)	0.997	**0.78 (0.70–0.87)**	**< 0.001**	1.05 (0.94–1.17)	0.391
Racism (ref. never)						
Daily	1.99 (0.94–4.19)	0.071	**0.78 (0.67–0.90)**	**< 0.001**	1.00 (0.87–1.16)	0.974
Weekly	1.00 (0.61–1.63)	0.983	**0.86 (0.78–0.94)**	**< 0.001**	0.96 (0.87–1.05)	0.311
Monthly	1.04 (0.69–1.58)	0.848	0.96 (0.87–1.07)	0.485	1.01 (0.91–1.11)	0.860
Unauthorized distribution of sensitive material (ref. never)						
Daily	1.72 (0.72–4.10)	0.212	**0.72 (0.62–0.84)**	**< 0.001**	1.04 (0.89–1.21)	0.654
Weekly	1.53 (0.99–2.37)	0.055	**0.84 (0.76–0.93)**	**< 0.001**	0.97 (0.89–1.07)	0.578
Monthly	1.25 (0.86–1.81)	0.245	0.95 (0.87–1.04)	0.248	1.03 (0.94–1.12)	0.537
Phishing attempts (ref. never)						
Daily	1.92 (0.82–4.54)	0.134	**0.75 (0.64–0.88)**	**< 0.001**	0.86 (0.72–1.01)	0.062
Weekly	1.15 (0.69–1.89)	0.598	**0.82 (0.73–0.91)**	**< 0.001**	0.93 (0.83–1.05)	0.246
Monthly	1.13 (0.73–1.75)	0.579	0.98 (0.90–1.08)	0.738	0.96 (0.87–1.05)	0.318
Misinformation (ref. never)						
Daily	**2.24 (1.25–4.03)**	**0.007**	0.93 (0.80–1.07)	0.298	0.91 (0.78–1.05)	0.195
Weekly	1.48 (0.93–2.34)	0.099	0.96 (0.86–1.07)	0.475	0.93 (0.83–1.04)	0.203
Monthly	1.18 (0.71–1.98)	0.525	1.07 (0.94–1.21)	0.318	0.94 (0.83–1.06)	0.304
Sale or distribution of drugs (ref. never)						
Daily	**1.85 (1.03–3.35)**	**0.041**	**0.68 (0.59–0.78)**	**< 0.001**	**1.19 (1.05–1.36)**	**0.009**
Weekly	1.57 (0.95–2.58)	0.076	**0.79 (0.72–0.87)**	**< 0.001**	1.05 (0.95–1.16)	0.331
Monthly	1.07 (0.66–1.72)	0.796	**0.85 (0.76–0.96)**	**0.006**	1.11 (0.99–1.23)	0.071
Harmful social media challenges (ref. never)						
Daily	1.51 (0.61–3.77)	0.359	**0.65 (0.55–0.75)**	**< 0.001**	1.13 (0.97–1.32)	0.111
Weekly	1.34 (0.87–2.06)	0.180	**0.87 (0.79–0.95)**	**0.003**	0.98 (0.89–1.07)	0.596
Monthly	**1.76 (1.21–2.57)**	**0.003**	0.95 (0.87–1.04)	0.293	0.97 (0.89–1.06)	0.479
Content that causes appearance pressures (ref. never)						
Daily	**1.81 (1.00–3.29)**	**0.050**	**0.63 (0.55–0.71)**	**< 0.001**	1.00 (0.89–1.13)	0.997
Weekly	**1.67 (1.05–2.65)**	**0.029**	**0.79 (0.71–0.88)**	**< 0.001**	0.94 (0.85–1.04)	0.236
Monthly	**2.63 (1.66–4.18)**	**< 0.001**	**0.86 (0.76–0.97)**	**0.014**	0.95 (0.85–1.06)	0.312

Fixed-effect multinomial logistic regression models: odds ratios (OR); 95% confidence intervals (CI); *ref.* reference category

The significance level was set at *p* < 0.05. The significant associations have been **bolded**

Regression models for each social media threat were run separately. The models were adjusted for gender, age, emotional intelligence, FAS, family support, friend support, PSMU, online communication with strangers. Social media threats were treated as outcome variables in the models

Emotional intelligence was only included for 15-year-olds

### The association of PSMU and online communication with strangers with social media threats

Adolescents with PSMU were more likely to report *daily* (ORs 3.00–5.66) and *weekly* (ORs 1.58–4.72) exposure to every social media threat except misinformation (Table 4). For example, problematic users were more than five times as likely to report *daily* exposure to cyberbullying (OR = 5.64, CI 95% = 2.97–10.69) and sexual harassment (OR = 5.66, CI 95% = 3.07–10.42). Those who reported intensive online communication with strangers were more likely to encounter eight out of the nine social media threats *daily* (ORs 2.03–6.02), as well as exposure to four social media threats *weekly* (ORs 1.96–2.91).

**Table 4:** The association of PSMU and online communication with strangers with social media threats

Variable	Problematic social media use (ref. non-problematic use)		Online communication with strangers (ref. non-intensive communication)	
	OR (*CI* 95%)	*p* value	OR (*CI* 95%)	*p* value
Cyberbullying (ref. never)				
Daily	**5.64 (2.97–10.69)**	**< 0.001**	**3.98 (1.59–10.00)**	**0.004**
Weekly	**4.72 (2.84–7.82)**	**< 0.001**	1.94 (0.96–3.95)	0.067
Monthly	**2.68 (1.71–4.20)**	**< 0.001**	1.17 (0.59–2.33)	0.658
Sexual harassment (ref. never)				
Daily	**5.66 (3.07–10.42)**	**< 0.001**	**5.82 (2.58–13.15)**	**< 0.001**
Weekly	**3.05 (1.83–5.08)**	**< 0.001**	**2.91 (1.60–5.28)**	**< 0.001**
Monthly	**1.97 (1.26–3.09)**	**0.003**	1.74 (0.84–3.60)	0.133
Racism (ref. never)				
Daily	**3.00 (1.77–5.10)**	**< 0.001**	**3.43 (1.77–6.68)**	**< 0.001**
Weekly	**1.83 (1.21–2.75)**	**0.004**	**2.32 (1.37–3.94)**	**0.002**
Monthly	1.08 (0.67–1.72)	0.763	1.14 (0.60–2.16)	0.686
Unauthorized distribution of sensitive material (ref. never)				
Daily	**4.58 (2.57–8.16)**	**< 0.001**	**4.36 (2.10–9.05)**	**< 0.001**
Weekly	**2.47 (1.62–3.77)**	**< 0.001**	1.85 (0.91–3.77)	0.089
Monthly	1.28 (0.81–2.02)	0.299	1.18 (0.61–2.25)	0.624
Phishing attempts (ref. never)				
Daily	**4.08 (2.22–7.49)**	**< 0.001**	**6.02 (2.93–12.36)**	**< 0.001**
Weekly	**3.08 (2.03–4.68)**	**< 0.001**	**2.52 (1.20–5.30)**	**0.017**
Monthly	1.36 (0.84–2.21)	0.209	1.72 (0.97–3.05)	0.064
Misinformation (ref. never)				
Daily	1.50 (0.77–2.92)	0.229	1.97 (0.82–4.76)	0.127
Weekly	1.05 (0.64–1.73)	0.846	1.00 (0.46–2.16)	0.997
Monthly	0.71 (0.39–1.32)	0.276	0.76 (0.33–1.76)	0.525
Sale or distribution of drugs (ref. never)				
Daily	**3.84 (2.19–6.73)**	**< 0.001**	**2.03 (1.01–4.10)**	**0.048**
Weekly	**2.40 (1.54–3.76)**	**< 0.001**	0.97 (0.53–1.79)	0.932
Monthly	1.75 (0.93–3.28)	0.081	0.58 (0.24–1.39)	0.218
Harmful social media challenges (ref. never)				
Daily	**3.85 (2.14–6.94)**	**< 0.001**	**3.18 (1.51–6.71)**	**0.002**
Weekly	**1.58 (1.04–2.39)**	**0.031**	**1.96 (1.12–3.43)**	**0.019**
Monthly	0.97 (0.61–1.54)	0.888	1.66 (0.92–2.99)	0.090
Content that causes appearance pressures (ref. never)				
Daily	**4.40 (2.55–7.60)**	**< 0.001**	**2.13 (1.12–4.06)**	**0.021**
Weekly	**2.86 (1.76–4.65)**	**< 0.001**	0.93 (0.49–1.77)	0.819
Monthly	**1.95 (1.07–3.55)**	**0.031**	1.08 (0.57–2.05)	0.808

Fixed-effect multinomial logistic regression models: odds ratios (OR); 95% confidence intervals (CI); *ref.* reference category

The significance level was set at *p* < 0.05. The significant associations have been **bolded**

Regression models for each social media threat were run separately. The models were adjusted for gender, age, emotional intelligence, FAS, family support, friend support, PSMU, online communication with strangers. Social media threats were treated as outcome variables in the models

Emotional intelligence was only included for 15-year-olds

### The association of social media threats with health

Adolescents who encountered any of the social media threats *daily* or *weekly* were more likely to report having poor self-rated health, frequent depressive feelings, and frequent anxiety symptoms, as compared to those who never reported such encounters. For instance, those exposed to misinformation *daily* were almost three times as likely to report poor self-rated health (OR = 2.83, CI 95% = 1.68–4.76), and approximately four times as likely to report frequent depressive feelings (OR = 4.15, CI 95% = 2.63–6.54) and frequent anxiety symptoms (OR = 3.78, CI 95% = 2.47–5.78; Table 5). Furthermore, adolescents who encountered any of the threats as infrequently as *once a month* were more likely to report having frequent depressive feelings than those who *never* experienced such threats (ORs 1.33–2.48). Similarly, adolescents with *monthly* exposure to eight out of the nine threats were more likely to report frequent anxiety symptoms (ORs 1.62–2.60). Adolescents exposed *monthly* to cyberbullying, sexual harassment, or phishing attempts were more likely to report poor self-rated health (ORs 1.54–1.97).

**Table 5:** The association of social media threats with health

Variable	Self-rated health (ref. good self-rated health)		Depressive feelings (ref. no frequent depressive feelings)		Anxiety (ref. no frequent anxiety)	
	OR (CI 95%)	*p* value	OR (CI 95%)	*p* value	OR (CI 95%)	*p* value
Cyberbullying (ref. never)						
Daily	**2.55 (1.31–4.97)**	**0.006**	**3.15 (1.69–5.85)**	**< 0.001**	**2.99 (1.59–5.61)**	**< 0.001**
Weekly	**3.20 (1.97–5.21)**	**< 0.001**	**2.75 (1.67–4.53)**	**< 0.001**	**3.63 (2.37–5.54)**	**< 0.001**
Monthly	**1.97 (1.35–2.88)**	**< 0.001**	**2.48 (1.79–3.43)**	**< 0.001**	**2.60 (1.84–3.66)**	**< 0.001**
Sexual harassment (ref. never)						
Daily	**3.14 (1.50–6.61)**	**0.004**	**4.08 (2.16–7.71)**	**< 0.001**	**3.62 (2.05–6.41)**	**< 0.001**
Weekly	**3.37 (2.24–5.08)**	**< 0.001**	**2.49 (1.66–3.73)**	**< 0.001**	**3.07 (2.10–4.50)**	**< 0.001**
Monthly	**1.54 (1.06–2.24)**	**0.023**	**2.22 (1.64–3.01)**	**< 0.001**	**2.34 (1.70–3.23)**	**< 0.001**
Racism (ref. never)						
Daily	**2.53 (1.57–4.09)**	**< 0.001**	**4.42 (2.66–7.33)**	**< 0.001**	**3.47 (2.26–5.34)**	**< 0.001**
Weekly	**1.98 (1.41–2.79)**	**< 0.001**	**2.87 (2.13–3.86)**	**< 0.001**	**2.99 (2.27–3.94)**	**< 0.001**
Monthly	1.01 (0.70–1.45)	0.977	**1.47 (1.10–1.97)**	**0.010**	**1.62 (1.25–2.11)**	**< 0.001**
Unauthorized distribution (ref. never)						
Daily	**3.32 (1.97–5.59)**	**< 0.001**	**3.51 (2.16–5.72)**	**< 0.001**	**3.12 (1.94–5.04)**	**< 0.001**
Weekly	**2.38 (1.71–3.32)**	**< 0.001**	**2.19 (1.62–2.97)**	**< 0.001**	**3.57 (2.59–4.92)**	**< 0.001**
Monthly	1.35 (0.96–1.91)	0.084	**1.53 (1.13–2.09)**	**0.007**	**1.65 (1.25–2.18)**	**< 0.001**
Phishing attempts (ref. never)						
Daily	**3.81 (2.10–6.88)**	**< 0.001**	**3.37 (1.99–5.72)**	**< 0.001**	**4.34 (2.62–7.18)**	**< 0.001**
Weekly	**3.06 (2.10–4.46)**	**< 0.001**	**2.61 (1.85–3.69)**	**< 0.001**	**3.04 (2.18–4.24)**	**< 0.001**
Monthly	**1.89 (1.34–2.67)**	**< 0.001**	**1.33 (1.01–1.75)**	**0.044**	**1.73 (1.30–2.30)**	**< 0.001**
Misinformation (ref. never)						
Daily	**2.83 (1.68–4.76)**	**< 0.001**	**4.15 (2.63–6.54)**	**< 0.001**	**3.78 (2.47–5.78)**	**< 0.001**
Weekly	**1.93 (1.22–3.05)**	**0.005**	**2.53 (1.73–3.69)**	**< 0.001**	**2.72 (1.97–3.74)**	**< 0.001**
Monthly	1.43 (0.87–2.36)	0.159	**1.54 (1.02–2.31)**	**0.040**	1.33 (0.93–1.90)	0.121
Sale or distribution of drugs (ref. never)						
Daily	**2.02 (1.26–3.24)**	**0.004**	**3.20 (2.17–4.73)**	**< 0.001**	**3.94 (2.72–5.70)**	**< 0.001**
Weekly	**1.80 (1.26–2.58)**	**0.001**	**1.86 (1.38–2.52)**	**< 0.001**	**2.75 (2.06–3.67)**	**< 0.001**
Monthly	1.16 (0.77–1.75)	0.479	**1.43 (1.02–1.99)**	**0.038**	**1.62 (1.17–2.24)**	**0.004**
Harmful social media challenges (ref. never)						
Daily	**2.13 (1.24–3.66)**	**0.007**	**4.18 (2.56–6.84)**	**< 0.001**	**4.58 (2.70–7.77)**	**< 0.001**
Weekly	**1.65 (1.13–2.42)**	**0.011**	**2.42 (1.80–3.26)**	**< 0.001**	**2.81 (2.09–3.76)**	**< 0.001**
Monthly	1.11 (0.80–1.55)	0.524	**1.59 (1.18–2.13)**	**0.002**	**1.80 (1.39–2.34)**	**< 0.001**
Content that causes appearance pressures (ref. never)						
Daily	**5.12 (3.39–7.74)**	**< 0.001**	**8.89 (6.21–12.73)**	**< 0.001**	**6.96 (4.85–9.97)**	**< 0.001**
Weekly	**2.14 (1.48–3.10)**	**< 0.001**	**3.32 (2.46–4.48)**	**< 0.001**	**4.94 (3.72–6.55)**	**< 0.001**
Monthly	0.98 (0.61–1.57)	0.925	**1.65 (1.17–2.33)**	**0.004**	**2.02 (1.50–2.73)**	**< 0.001**

Fixed-effect multinomial logistic regression models: odds ratios (OR); 95% confidence intervals (CI); *ref.* reference category

The significance level was set at *p* < 0.05. The significant associations have been **bolded**

Regression models for each social media threat were run separately. Health outcomes were treated as outcome variables in the models

The models were adjusted for gender, age, FAS

## Discussion

The study investigated the prevalence among adolescents of nine social media threats, the associations of individual and social factors, PSMU, and online communication with strangers with the nine threats, and the association of such threats with health.

We expected the prevalence of exposure to different social media threats to vary among adolescents, depending on the threat type and the reporting frequency (H1). This hypothesis was confirmed by the findings. At a *daily* level, the most common social media threats were misinformation (12.9%) and content causing appearance pressures (9.1%), and at a *weekly* level misinformation (44.2%), harmful social media challenges (22.3%), and unauthorized distribution of sensitive material (22.2%). Exposure to misinformation was the threat least often expressed as ‘never encountered’ (17.3%). The findings of our study are in line with previous research indicating that, in particular, misinformation has rapidly proliferated in adolescent social media [14]. Our findings also show the unauthorized distribution of sensitive material to be more common than previously reported [59]. Furthermore, our results shed new light on the prevalence of harmful or dangerous social media challenges among adolescents, bearing in mind that previous studies have focused on adults [43], or on harmful content (but not in the form of challenges) [8], or else have been limited to specific platforms [103] or specific challenges [104]. It should be borne in mind that the prevalence of social media threats per se is not the sole indicator of the harmfulness of threats for adolescents. For instance, cyberbullying and sexual harassment, reported on a daily or a weekly basis by 9.6% (for cyberbullying) and 10.6% (for sexual harassment), are inherently more serious threats compared to, for instance, misinformation [74]. Both cyberbullying and sexual harassment specifically target the individual recipient of the message, while several other threats can to some extent affect anyone who comes across the message. Nevertheless, given that vulnerabilities beget vulnerabilities among adolescents [6], it is likely that social media threats co-occur, and that certain adolescents face many threats simultaneously; hence, the possibility of widening disparities should be considered [see 105].

We expected that individual and social factors, PSMU, and online communication with strangers would differently explain exposure to various social media threats (H2) This hypothesis was also confirmed. In line with previous studies [51], we found that girls were more likely to encounter content causing appearance pressures. Boys, on the other hand, were more likely to report daily exposure to seven out of the nine social media threats (e.g., cyberbullying, racism, phishing attempts).

Based on suggestions by scholars [15, 54] we studied adolescents’ social media threats through a developmental lens and found older adolescents (13- and 15-year-olds) to be more likely than 11-year-olds to encounter social media threats, daily, weekly, and monthly (with some exceptions). One reason for this could be that older adolescents have had more years to experiment with social media, and they use social media more intensively [92, 94].

We further hypothesized that individual (e.g., emotional intelligence) and social (e.g., family support) factors could protect adolescents from encountering social media threats (H2.1). Our findings showed that these factors do indeed have a potential to mitigate adolescent exposure to social media threats. For example, a higher level of *emotional intelligence* was linked to less likely daily exposure to cyberbullying, sexual harassment, and phishing attempts, thus highlighting the importance of emotional skills as a protection against social media threats. A similar notion could apply to the role of *family support*, since a higher level of family support was negatively associated with daily and weekly exposure to all other social media threats, apart from misinformation. The promotion of supportive parent-child relationships, as opposed to the adoption of overly restrictive parental monitoring strategies, could encourage adolescent disclosure, and thus lead to more positive outcomes [106].

The role of *friend support* on social media threats was more complex and it varied across the social media threats. On the one hand, higher friend support was positively associated with daily exposure to the sale and distribution of drugs. On the other hand, higher friend support had a negative association with daily exposure to cyberbullying. Previous literature indicates that the social media context may amplify peer influence processes, which affect adolescent behaviours and cognitions [41]. As an example, in adolescence, peer groups are approached and valued to a significant degree, and through drug-related behaviour adolescents may try to connect with deviant peers and enhance their social status [42]. However, the same friendships that amplify adolescent risk behaviour through social media may simultaneously work as a barrier against other threats such as cyberbullying [75].

It was also hypothesized that certain factors describing how adolescents use social media (notably PSMU and intensive online communication with strangers), were among the factors placing adolescents in a vulnerable situation regarding social media threats (H2.2). Adolescents with PSMU and those reporting intensive communication with strangers, were in fact more likely to report daily exposure to every social media threat, with the exception of misinformation. Similarly, there was support for the claim that vulnerabilities tend to beget vulnerabilities [see 6] including in the digital environment (involving the co-occurrence of PSMU and social media threats). Furthermore, although previous research has shown social media solicitation (i.e., approaching young people with ill intentions) to be rare, our results show that adolescents engaging in intensive communication with strangers are at greater risk of encountering various threats. Adolescents should thus be provided with the knowledge and skills to operate with people they do not know, and identify malicious intents on social media.

Exposure to various social media threats was further expected to explain negative health among adolescents, with the associations varying between different social media threats and the prevalence of the exposure (H3). This study showed that daily and weekly exposure to social media threats was systematically associated with poor self-rated health, and with frequent depressive feelings and anxiety symptoms (thus confirming our third hypothesis). Moreover, exposure to any of the nine social media threats as seldom as once a month increased the likelihood of at least one negative health outcome. There were also threats (notably cyberbullying and sexual harassment) to which monthly exposure increased the likelihood of all the studied negative health outcomes. In general terms, the odds ratios for experiencing negative mental health increased when the frequency of exposure to social media threats increased. For instance, monthly exposure to harmful social media challenges increased the likelihood of frequent depressive feelings by 59%, whereas daily exposure to such challenges increased the likelihood by 318% as compared to those who were never exposed to harmful challenges. Such findings are consistent with previous research indicating increased exposure to online risk as a contributor to negative health outcomes [107, 108].

However, exceptions also emerged. For example, the association between exposure to cyberbullying and poor self-rated health was strongest among those who reported bullying weekly as opposed to daily. Such findings may have to do with the type of bullying (i.e., which form has the most severe health effect) [109], and whether those who self-report daily cyberbullying experience less severe forms of aggressive online behaviour, and hence less severe consequences for health. Consequently, more nuanced research is needed, given that substantial within-threat variation could exist in the social media threats explored.

The associations between social media threats and negative health among adolescents raise important questions from an intervention and policy-making perspective, regarding how threats should be prioritized, and at which threats limited resources should be targeted. For example, researchers [14] have identified misinformation as a clear public health challenge, especially due to the co-occurrence of persistent health disparities– yet, as discussed above, social media threats should not be evaluated purely by the prevalence of exposure. In this regard, it is worth noting that while 79.5% of adolescents had never encountered cyberbullying and 77.7% had never encountered sexual harassment, even one encounter with such a situation could be detrimental to adolescent health. This is especially the case, insofar as this and previous studies have systematically shown an association between such threats and negative health indicators [9, 110].

### Strengths and limitations

The present study had several strengths, including a large-scale nationally representative sample of adolescents and the use of validated instruments, plus a carefully considered distinction between different social media threats. Furthermore, to our knowledge, this is the first study to provide evidence on the association between PSMU and several other threats (e.g., harmful social media challenges), thus, opening up novel horizons for future studies and interventions. However, the findings should be interpreted with several limitations in mind. Firstly, the study’s cross-sectional design does not allow causal inferences. Secondly, it can be argued that self-reporting instruments may not give an objective view of adolescent exposure to social media threats, and that self-reporting measures of media experiences may not be a legitimate substitute for more objective measures [111]. For example, in terms of cyberbullying, the power imbalance between the perpetrator and the victim cannot be precisely measured via the type of self-reporting instrument used in this study. For instance, researchers argue that power might behave differently in the online context and that power dynamics can manifest through technological proficiency or possession of harmful content [112, 113]. Hence, it could be interpreted that any individual who can exploit technology to harm others holds a position of power, at least temporarily, in relation to the victim of the assault [113]. Similarly, self-reporting instruments may not provide an objective reflection of certain individual and social factors, given that not all individuals will necessarily perceive, for instance, emotional intelligence in the same way. However, the information given by self-reporting instruments is necessary if one is to explore individuals’ personal experiences and views [114] on their emotional intelligence. One must also bear in mind that experiences of social media threat exposure are individual and subjective (as in the case of cyberbullying); hence, they need to be investigated via measures considering individual experiences (as in this study). Nonetheless, the information could have been enriched by the views of multiple informants, including peers, parents, or teachers.

Finally, one must be cautious about generalizing the results beyond the study populations (e.g., to non-white, and low-income countries). To overcome these limitations, future studies should employ cross-national study settings, wider study populations, and longitudinal research. Furthermore, moderation and mediation approaches could be applied to better understand (i) the factors protecting against social media threats, and (ii) how social media threats operate in the association between the intensity of social media use and health, and in the associations between various social media activities and health outcomes. There is also a need to investigate how social media threats co-occur and interrelate, for example, whether being exposed to one social media threat increases the likelihood of being exposed to many. One could also seek to determine whether certain types of threats accumulate for specific individuals, and how the individuals themselves act or react (e.g., as regards cyberbullying perpetration, sexual harassment perpetration, and the sharing of misinformation) on social media. Person-oriented approaches such as Latent Class Analysis (LCA) would be advisable in this regard.

### Conclusions

Our study indicates that intervention and health promotion efforts are needed to reduce adolescent exposure to social media threats and associated negative health outcomes. The efforts should consider the individual and social differences among adolescents (the aim being to promote equity by ensuring that adolescents in vulnerable situations benefit proportionately more from such efforts) [see 105]. The measures taken could aim to support resources such as emotional intelligence and family support against social media threats. Furthermore, we suggest that, in particular, governments and service providers should act and collaborate to reduce adolescent encounters with social media threats. The negative impacts of social media threats on health could be mitigated by directing resources to vulnerable populations, utilizing both algorithmic strategies and caregiver interventions [115]. Additionally, the use of advanced technologies such as natural language processing and data mining can aid in identifying and removing online content that is harmful, provocative, or lacking scientific validity [115]. It is also important to keep in mind the positive aspects of social media use, including the increased opportunities it allows for social connection [5]. Altogether, efforts to ensure safe social media for adolescents are crucial, as highlighted also by the development strategies undertaken in Europe [24, 25].

## Data Availability

The dataset supporting the conclusions of this article will be available in the HBSC Data Management Centre repository: https://www.uib.no/en/hbscdata/113290/open-access. The 2022 data will be made available in 2026 according to the HBSC protocol. The analysis path and the code will be made available in OSF: https://osf.io/ upon acceptance of the manuscript.
